# Knowledge and attitudes toward artificial intelligence in nursing among various categories of professionals in China: a cross-sectional study

**DOI:** 10.3389/fpubh.2024.1433252

**Published:** 2024-07-02

**Authors:** Xiaoyan Wang, Fangqin Fei, Jiawen Wei, Mingxue Huang, Fengling Xiang, Jing Tu, Yaping Wang, Jinhua Gan

**Affiliations:** ^1^School of Nursing, Southwest Medical University, Luzhou, Sichuan Province, China; ^2^Department of Ophthalmology, The Affiliated Hospital, Southwest Medical University, Luzhou, Sichuan, China; ^3^Department of Nursing, First People’s Hospital of Huzhou, Huzhou University, Huzhou, Zhejiang Province, China; ^4^Department of Nursing, Shenzhen Eye Institute, Shenzhen Eye Hospital, Jinan University, Shenzhen, Guangdong Province, China

**Keywords:** artificial intelligence, nursing, knowledge, attitude, concern, ethic

## Abstract

**Objectives:**

The application of artificial intelligence (AI) in healthcare is an important public health issue. However, few studies have investigated the perceptions and attitudes of healthcare professionals toward its applications in nursing. This study aimed to explore the knowledge, attitudes, and concerns of healthcare professionals, AI-related professionals, and others in China toward AI in nursing.

**Methods:**

We conducted an online cross-sectional study on nursing students, nurses, other healthcare professionals, AI-related professionals, and others in China between March and April 2024. They were invited to complete a questionnaire containing 21 questions with four sections. The survey followed the principle of voluntary participation and was conducted anonymously. The participants could withdraw from the survey at any time during the study.

**Results:**

This study obtained 1,243 valid questionnaires. The participants came from 25 provinces and municipalities in seven regions of China. Regarding knowledge of AI in nursing, 57% of the participants knew only a little about AI, 4.7% did not know anything about AI, 64.7% knew only a little about AI in nursing, and 13.4% did not know anything about AI in nursing. For attitudes toward AI in nursing, participants were positive about AI in nursing, with more than 50% agreeing and strongly agreeing with each question on attitudes toward AI in nursing. Differences in the numbers of participants with various categories of professionals regarding knowledge and attitudes toward AI in nursing were statistically significant (*p* < 0.05). Regarding concerns and ethical issues about AI in nursing, every participant expressed concerns about AI in nursing, and 95.7% of participants believed that it is necessary to strengthen medical ethics toward AI in nursing.

**Conclusion:**

Nursing students and healthcare professionals lacked knowledge about AI or its application in nursing, but they had a positive attitude toward AI. It is necessary to strengthen medical ethics toward AI in nursing. The study’s findings could help develop new strategies benefiting healthcare.

## 1 Introduction

Artificial intelligence (AI) is a branch of computer science. It is an intelligent system that uses computer technology to simulate, extend, expand, and realize the human mind to carry out thinking activities, learn knowledge, etc., and to help human beings solve problems (1, 2). With the rapid development of the Internet and AI, the combination of AI and medicine has had far-reaching effects on healthcare systems and nursing (3–7). AI plays a significant role in clinical care, nursing education, nursing management, etc.

In clinical care, AI technology, especially AI robots, has a relatively wide range of applications throughout the entire process from patient admission to discharge, and even post-discharge home rehabilitation care. In busy hospital outpatient clinics, mobile intelligent guide robots play an important role in optimizing outpatient services, improving outpatient patient’s experience, and reducing the workload of nurses (8). In hospital intensive care unit, robots with different functions such as logistics and disinfection robots, rehabilitation assistance robots, and treatment assistance robots contribute to reduce the workload of nurses and provide personalized needs (9). When caring for infected patients undergoing isolation, nurses face the risk of infection at any time. The emergence of mobile robots not only reduces the risk of infection, but also improves the efficiency and quality of care (10). For discharged patients with disabilities or walking disabilities, compared with walking aids, mobility robots can sense the surrounding environment and the user’s intention, flexibly assisting walking and reducing the occurrence of adverse events (11, 12). Exoskeleton robots can help stroke patients improve their gait and facilitate their rehabilitation (13). In addition, socially assistive robots can promote social interactions among home-bound older adults, reduce their sense of loneliness, and enhance their sense of well-being (14).

Generative AI is more widely used in nursing education. Generative AI is a class of AI models including natural language processing, machine learning, reasoning and decision-making and other multi-disciplinary technologies, which can creatively generate images, text, phonetics, etc., and has a promising application in the field of nursing education. Currently, ChatGPT is one of the most powerful generative AI models, and its use not only meets the personalized learning needs of nursing students, but also improves the efficiency of teachers and promotes collaboration and communication between teachers and students (15). ChatGPT simulates learning environments or hospital scenarios for nursing students through virtual reality, which is conducive to improving the students’ confidence and learning ability (16, 17). In addition, ChatGPT can provide nursing students with timely learning feedback, meet the need for rapid access to information, and improve time management skills (18, 19).

Reducing the occurrence of adverse events such as falls and pressure injury in hospitals is an important part of nursing management. Based on AI algorithms such as machine learning that can process large amounts of data and construct predictive models with good performance, it can help nursing managers to formulate appropriate management plans, while nurses can effectively identify risk factors for pressure injury and falls, and formulate nursing care measures to address the risk factors to reduce the incidence rate (20, 21). In addition, predictive models based on machine learning algorithms have also been used to predict unplanned readmissions of patients and diseases to reduce healthcare-related costs through active intervention (22, 23).

Artificial intelligence’s application in nursing demonstrates its unique advantages. It can help optimize nursing procedures, improve nursing practice efficiency, and facilitate precision nursing (24). AI can efficiently analyze large amounts of complex data to help diagnose various medical conditions and reduce the workload of healthcare workers (25); disease risk prediction models built based on machine-learning algorithms can quickly identify diseases (26). Nevertheless, AI’s application in nursing has drawbacks and raises ethical issues (24).

Many countries have studied the perceptions and attitudes of medical students, healthcare professionals, and patients toward the use of AI in the medical field (27–30). Many studies have demonstrated positive attitudes toward AI; in contrast, controversial perceptions regarding AI have also been reported (31, 32). However, few studies have investigated the perceptions and attitudes of medical students and healthcare workers toward AI’s application in nursing and reported surveys on ethical research. Because the development of AI in nursing is still in its infancy, advantages and problems exist in the field. This study aimed to investigate the perceptions, attitudes, and concerns of nursing students and healthcare professionals on AI in nursing and their views on ethical research, which could provide insight into developing strategies for improving AI’s application in the nursing field.

## 2 Methods

### 2.1 Study design and participants

We conducted an online cross-sectional study involving nursing students, nurses, physicians, other professionals, and technicians in China from March to April 2024. The inclusion criteria were as follows: (a) voluntary participation in the study and (b) inclusion of undergraduate nursing students, graduate nursing students, nurses, physicians, AI-related professionals, other professionals, and technical staff. The exclusion criteria were as follows: (a) those who were not willing to participate in the study or who withdrew during the study, (b) those who filled out the questionnaire incompletely, and (c) participants aged <18.

The questionnaire developed for this study consisted of 21 questions. The sample size was 5–10 times the number of variables, and 126–252 participants were included, considering a 20% invalid sample rate. The study included 1,243 participants. Informed consent was obtained from all participants.

### 2.2 Questionnaire development

After reviewing the literature on AI knowledge and attitudes, we synthesized questionnaires with good reliability and validity (33–35). The questionnaire was initially developed in English and then translated into Chinese. Before distributing the questionnaire, we combined the suggestions of experts and pre-survey participants to modify and improve the questionnaire and finally generated the official questionnaire. This was followed by a pilot study in which the questionnaire was used to conduct an exploratory factor analysis of 50 participants to clarify its reliability and validity. The reliability analysis results of the questionnaire were expressed using Cronbach’а coefficient, and the validity analysis results were expressed using Kaiser-Meyer-Olkin (KMO) and Bartlett’s test of sphericity (36). The Cronbach’а coefficient of the attitude toward the Nursing AI part of the questionnaire was 0.860, indicating good reliability. The KMO value of the attitude toward the Nursing AI questionnaire was 0.768, and the significance of Bartlett’s test of sphericity was less than 0.001, indicating that attitudes toward AI in the nursing section of the questionnaire were suitable for factor analysis. Exploratory factor analysis using principal component analysis revealed that the loadings squared and variance accumulated at a factor number of two reached 65.79% with good interpretability. The rotated component matrix is shown in Appendix Table 1, which showed good structural validity of the questionnaire. The sections on knowledge, concerns, and ethical research about AI were not suitable for questionnaire reliability tests because of the small number of questions and the inclusion of multiple-choice questions. Therefore, only attitudes toward AI in nursing were tested for reliability and validity.

### 2.3 Data collection

The questionnaire contained 21 questions in four sections. The first part of the questionnaire consisted of sociodemographic information, including six questions on sex, birth year and month, place of residence, various categories of professionals, educational backgrounds, and professional title. The second part of the questionnaire was based on the knowledge of AI or AI in nursing. It contained three questions and was conducted using a four-point Likert classification. The third part of the questionnaire focused on attitudes toward AI in nursing and consisted of nine questions rated on a four-point Likert scale. The fourth part of the questionnaire, which concerned ethical reviews about AI in nursing, contained three questions: two multiple-choice questions and one single-choice question.

### 2.4 Procedure

This survey was based on the Questionnaire Star App (China’s professional questionnaire survey application), which is easy to edit and distribute. The purpose and significance of this survey were explained to the participants in detail before its release. The collected information will be used for academic research only. The survey followed the principle of voluntary participation and was conducted anonymously. The participants could withdraw from the study at any time during the survey. Snowball sampling was used for data collection. The data collected using the Questionnaire Star App were imported into Microsoft Excel 2021 for organization and analysis.

According to Article 3 of the Measures for Ethical Review of Biomedical Research Involving Humans issued by the National Health and Family Planning Commission in 2016, no ethical review was required for this study (35).

### 2.5 Statistical analyses

SPSS (version 27.0) was used for data analysis. Categorical variables were expressed as frequencies and percentages, and continuous variables were expressed as means and standard deviations. The correlation between the categorical variables was tested using the Chi-square test and ratio analysis, and a *p* value of less than 0.05 indicated statistical significance.

## 3 Results

From 1,277 participants who participated in the survey, we obtained 1,243 valid questionnaires (97.34%) and 34 invalid questionnaires where age did not meet the inclusion criteria. Participants came from 25 provinces and municipalities in seven regions in China, including Southwest China (Sichuan, Guizhou, Yunnan, and Chongqing), North China (Beijing, Tianjin, Hebei, and Shanxi), Northeast China (Jilin and Inner Mongolia Autonomous Region), East China (Shanghai, Jiangsu, Zhejiang, Anhui, Jiangxi, Shandong, and Fujian), Central China (Hubei and Henan), South China (Guangdong and Guangxi Zhuang Autonomous Region), and Northwest China (Shaanxi, Gansu, and Xinjiang Uygur Autonomous Region).

### 3.1 Socio-demographic characteristics

In this survey, females accounted for 78.2% of all participants; most participants were 20–40 years old, accounting for 66.34%. The largest numbers of participants included those who lived in prefecture-level cities (36.8%), nurses (30.4%) who were followed by undergraduate nursing students (27.3%), those with a Bachelor’s degree (57.8%), and those without a professional title (45.5%) (Table 1).

**Table 1:** Baseline characteristics of the participants.

		Number	Percentage (%)
Gender	Male	271	21.80%
	Female	972	78.20%
Age(Y, years)	Y < 20	230	18.50%
	20 ≤ Y<40	824	66.30%
	40 ≤ Y<60	187	15%
	Y ≥ 60	2	0.20%
Place of residence	Provincial capital city or first-tier city	427	34.40%
	Prefecture-level city	457	36.80%
	County and below	346	27.80%
	Other	13	1.00%
Various categories of professionals	Undergraduate nursing student	339	27.30%
	Postgraduate nursing student	102	8.20%
	Nurse	378	30.40%
	Medical professional or technician (non-nursing)	154	12.40%
	Artificial intelligence related professional	134	10.80%
	Other professional	136	10.90%
Educational backgrounds	College or less	258	20.80%
	Bachelor’s degree (or bachelor’s degree in progress)	718	57.80%
	Master’s degree (or master’s degree in progress)	217	17.50%
	Doctoral degree or higher (or doctoral degree in progress)	50	4.00%
Professional title level	Ungraded	566	45.50%
	Junior	293	23.60%
	Intermediate	269	21.60%
	Senior	115	9.30%

### 3.2 Knowledge of AI

**Figure 1:**
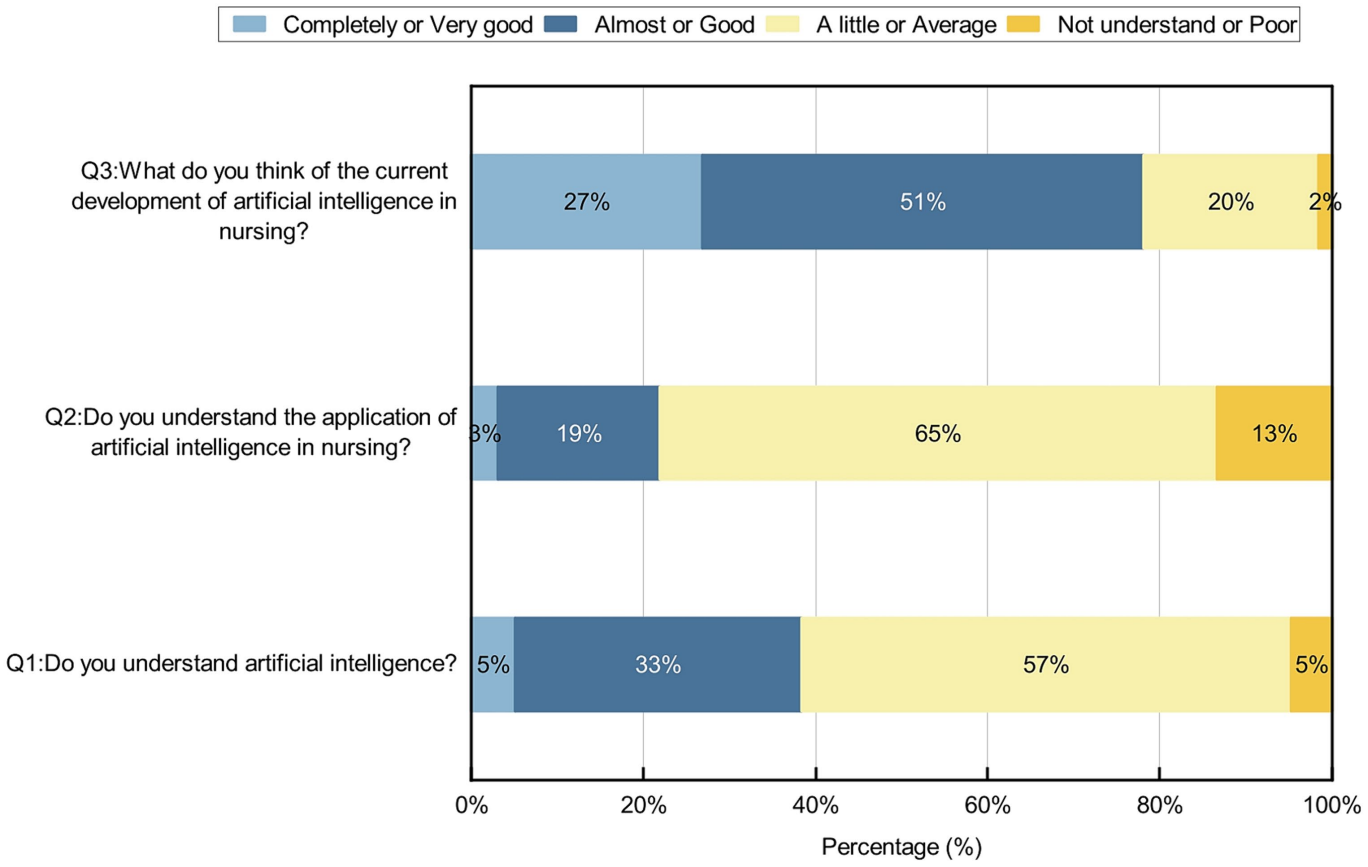
Participants’ knowledge of AI in nursing.

**Figure 2:**
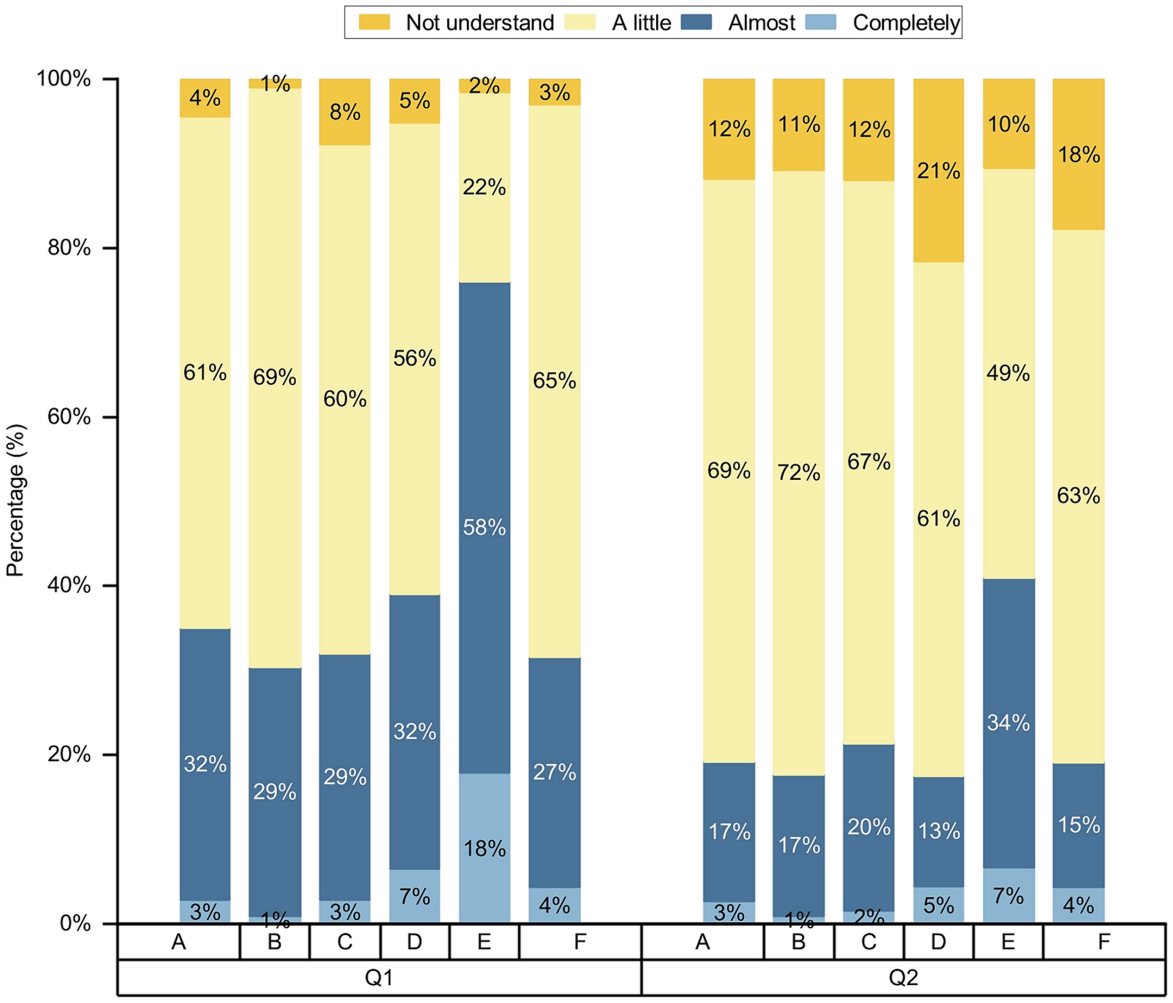
Comparison of differences in knowledge toward AI in nursing among participants with various categories of professionals. **(A)** Undergraduate nursing students; **(B)** postgraduate nursing students; **(C)** nurses; **(D)** other healthcare professionals; **(E)** AI-related professionals; and **(F)** other professionals; Q1-Q2, the questions of knowledge of AI in nursing.

Participants were not sufficiently aware of AI, and their knowledge of AI needed to be improved. Of the participants, 57% knew only a little about AI, 4.7% did not know anything about AI, 64.7% knew only a little about AI in nursing, and 13.4% did not know anything about AI in nursing; 26.9 and 51.2% of the participants believed that the application of AI to nursing is very good and good, respectively (Figure 1). We conducted chi-square test for gender, age, place of residence, various categories of professionals, educational backgrounds and professional title level, and the results showed that different genders, ages, place of residences, and various categories of professionals, educational backgrounds on the knowledge of AI and AI in nursing are all *p* values less than 0.05; the *p* values of different ages, place of residences, and educational backgrounds on the development trend of AI in nursing are less than 0.05, and the *p* values of different genders and categories of participants on the development trend of AI in nursing are greater than 0.05, as shown on Tables 2–6 and Figure 2. In addition, *p* values of different professional title level of nurses on the knowledge of AI and on the development trend of AI in nursing are greater than 0.05, as shown on (Table 7).

**Table 2:** Knowledge and attitudes toward AI in nursing across different genders of participants.

		Male	Female	Chi-square	*p* value
Q1: Do you understand artificial intelligence?	Completely	30(48.4%)	32(51.6%)	64.522	<0.001
	Almost	125(30.2%)	289(69.8%)		
	A little	111(15.7%)	597(84.3%)		
	Not understand	5(8.5%)	54(91.5%)		
Q2: Do you understand the application of artificial intelligence in nursing?	Completely	16(42.1%)	22(57.9%)	14.938	0.002
	Almost	62(26.5%)	172(73.5%)		
	A little	156(19.4%)	648(80.6%)		
	Not understand	37(22.2%)	130(77.8%)		
Q3: What do you think of the current development of artificial intelligence in nursing?	Very good	84(25.1%)	250(74.9%)	3.429	0.33
	Good	129(20.3%)	508(79.7%)		
	Average	53(20.9%)	200(79.1%)		
	Poor	5(26.3%)	14(73.7%)		
Q4: Do you agree that artificial intelligence will revolutionize the field of nursing?	Strongly agree	82(32%)	174(68.0%)	25.355	<0.001
	Agree	151(21%)	568(79%)		
	Disagree	31(13.7%)	196(86.3%)		
	Strongly disagree	7(17.7%)	34(82.9)		
Q5: Do you agree that the application of artificial intelligence in nursing can improve patients care?	Strongly agree	102(27%)	276(73%)	8.691	0.034
	Agree	153(19.5%)	633(80.5%)		
	Disagree	11(19.3%)	46(80.7%)		
	Strongly disagree	5(22.7%)	17(77.3%)		
Q6: Do you agree that the application of artificial intelligence in nursing can improve nursing decision-making?	Strongly agree	93(30.7%)	210(69.3%)	19.613	<0.001
	Agree	154(19.5%)	636(80.5%)		
	Disagree	20(15.5%)	109(84.5%)		
	Strongly disagree	4(19.0%)	17(81.0%)		
Q7: Do you agree that artificial intelligence in nursing can improve the health of populations?	Strongly agree	89(29.1%)	217(70.9%)	14.32	0.003
	Agree	164(20.1%)	652(79.9%)		
	Disagree	15(15.0%)	85(85.0%)		
	Strongly disagree	3(14.3%)	18(85.7%)		
Q8: Do you agree that the application of artificial intelligence in nursing will reduce healthcare costs?	Strongly agree	101(31.7%)	218(68.3%)	31.589	<0.001
	Agree	147(20.7%)	579(79.8%)		
	Disagree	19(11.0%)	154(89.0%)		
	Strongly disagree	4(16.0%)	21(84.0%)		
Q9: Do you agree that the application of artificial intelligence will reduce the burden on healthcare workers?	Strongly agree	112(27.3%)	299(72.7%)	12.427	0.006
	Agree	140(18.5%)	615(81.5%)		
	Disagree	15(25.9%)	43(74.1%)		
	Strongly disagree	4(21.1%)	15(78.9%)		
Q10: Do you agree that artificial intelligence in nursing will change the role of nurses in the future?	Strongly agree	77(30.7%)	174(69.3%)	15.012	0.002
	Agree	144(20.0%)	575(80.0%)		
	Disagree	41(17.9%)	188(82.1%)		
	Strongly disagree	9(20.5%)	35(79.5%)		
Q11: Do you agree that artificial intelligence in nursing will replace the work of nurses?	Strongly agree	49(31.4%)	107(68.6%)	15.619	0.001
	Agree	118(23.7%)	380(76.3%)		
	Disagree	77(18.1%)	348(81.9%)		
	Strongly disagree	27(16.5%)	137(83.5%)		
Q12: Do you accept the application of artificial intelligence in nursing?	Strongly agree	103(26.4%)	287(73.6%)	14.711	0.002
	Agree	161(19.4%)	667(80.6%)		
	Disagree	2 (11.8%)	15(88.2%)		
	Strongly disagree	271 (21.8%)	972(78.2%)		

**Table 3:** Knowledge and attitudes toward AI in nursing among participants with different ages.

		Y < 20	20 ≤ Y<40	40 ≤ Y<60	Y ≥ 60	Chi-square	*p* value
Q1: Do you understand artificial intelligence?	Completely	7(11.3%)	49(79.0%)	6(9.7%)	0(0.0%)	17.996	0.032
	Almost	67(16.2%)	288(69.6%)	58(14.0%)	1(0.2%)		
	A little	150(21.2%)	449(63.4%)	108(15.3%)	1(0.1%)		
	Not understand	6(10.2%)	38(64.4%)	15(25.4%)	0(0.0%)		
Q2: Do you understand the application of artificial intelligence in nursing?	Completely	7(18.4%)	28(73.7%)	3(7.9%)	0(0.0%)	27.44	<0.001
	Almost	28(12.0%)	180(76.9%)	26(11.1%)	0(0.0%)		
	A little	170(21.1%)	513(63.8%)	119(14.8%)	2(0.2%)		
	Not understand	25(15.0%)	103(61.7%)	39(23.4%)	0(0.0%)		
Q3: What do you think of the current development of artificial intelligence in nursing?	Very good	43(12.9%)	228(68.3%)	62(18.6%)	1(0.3%)	28.291	<0.001
	Good	149(23.4%)	404(63.4%)	84(13.2%)	0(0.0%)		
	Average	1(5.3%)	15(78.9%)	3(15.8%)	0(0.0%)		
	Poor	230(18.5%)	824(66.3%)	187(15.0%)	2(0.2%)		
Q4: Do you agree that artificial intelligence will revolutionize the field of nursing?	Strongly agree	25(9.8%)	172(67.2%)	58(22.7%)	1(0.4%)	38.687	<0.001
	Agree	137(19.1%)	478(66.5%)	103(14.3%)	1(0.1%)		
	Disagree	59(26.0%)	149(65.6%)	19(8.4%)	0(0.0%)		
	Strongly disagree	9(22.0%)	25(61.0%)	7(17.1%)	0(0.0%)		
Q5: Do you agree that the application of artificial intelligence in nursing can improve patients care?	Strongly agree	51(13.5%)	251(66.4%)	75(19.8%)	1(0.3%)	27.254	<0.001
	Agree	163(20.7%)	523(66.5%)	99(12.6%)	1(0.1%)		
	Disagree	10(17.5%)	41(71.9%)	6(10.5%)	0(0.0%)		
	Strongly disagree	6(27.3%)	9(40.9%)	7(31.8%)	0(0.0%)		
Q6: Do you agree that the application of artificial intelligence in nursing can improve nursing decision-making?	Strongly agree	41(13.5%)	202(66.7%)	59(19.5%)	1(0.3%)	22.829	0.007
	Agree	166(21.0%)	515(65.2%)	108(13.7%)	1(0.1%)		
	Disagree	17(13.2%)	97(75.2%)	15(11.6%)	0(0.0%)		
	Strongly disagree	6(28.6%)	10(47.6%)	5(23.8%)	0(0.0%)		
Q7: Do you agree that artificial intelligence in nursing can improve the health of populations?	Strongly agree	44(14.4%)	201(65.7%)	60(19.6%)	1(0.3%)	25.959	0.001
	Agree	166(20.3%)	545(66.8%)	105(12.9%)	0(0.0%)		
	Disagree	13(13.0%)	69(69.0%)	17(17.0%)	1(1.0%)		
	Strongly disagree	7(33.3%)	9(42.9%)	5(23.8%)	0(0.0%)		
Q8: Do you agree that the application of artificial intelligence in nursing will reduce healthcare costs?	Strongly agree	38(11.9%)	219(68.7%)	62(19.4%)	0(0.0%)	27.963	<0.001
	Agree	140(19.3%)	482(66.4%)	102(14.0%)	2(0.3%)		
	Disagree	48(27.7%)	108(62.4%)	17(9.8%)	0(0.0%)		
	Strongly disagree	4(16.0%)	15(60.0%)	6(24.0%)	0(0.0%)		
Q9: Do you agree that the application of artificial intelligence will reduce the burden on healthcare workers?	Strongly agree	58(14.1%)	278(67.6%)	75(18.2%)	0(0.0%)	19.268	0.026
	Agree	157(20.8%)	497(65.8%)	99(13.1%)	2(0.3%)		
	Disagree	14(24.1%)	36(62.1%)	8(13.8%)	0(0.0%)		
	Strongly disagree	1(5.3%)	13(68.4%)	5(26.3%)	0(0.0%)		
Q10: Do you agree that artificial intelligence in nursing will change the role of nurses in the future?	Strongly agree	25(10.0%)	168(66.9%)	57(22.7%)	1(0.4%)	53.526	<0.001
	Agree	130(18.1%)	481(66.9%)	107(14.9%)	1(0.1%)		
	Disagree	65(28.4%)	151(65.9%)	13(5.7%)	0(0.0%)		
	Strongly disagree	10(22.7%)	24(54.5%)	10(22.7%)	0(0.0%)		
Q11: Do you agree that artificial intelligence in nursing will replace the work of nurses?	Strongly agree	16(10.3%)	111(71.2%)	29(18.6%)	0(0.0%)	21.62	0.006
	Agree	81(16.3%)	335(67.3%)	81(16.3%)	1(0.2%)		
	Disagree	96(22.6%)	269(63.3%)	60(14.1%)	0(0.0%)		
	Strongly disagree	37(22.6%)	109(66.5%)	17(10.4%)	1(0.6%)		
Q12: Do you accept the application of artificial intelligence in nursing?	Strongly agree	38(9.7%)	266(68.2%)	85(21.8%)	1(0.3%)	48.651	<0.001
	Agree	187(22.6%)	540(65.2%)	100(12.1%)	1(0.1%)		
	Disagree	3(17.6%)	13(76.5%)	1(5.9%)	0(0.0%)		
	Strongly disagree	230(18.5%)	824(66.3%)	187(15.0%)	2(0.2%)		

**Table 4:** Knowledge and attitudes toward AI in nursing among participants with different places of residence.

		Provincial capital city or first-tier city	Prefecture-level city	County and below	Other	Chi-square	*p* value
Q1: Do you understand artificial intelligence?	Completely	36(58.1%)	12(19.4%)	13(21.0%)	1(1.6%)	35.611	<0.001
	Almost	149(36.0%)	166(40.1%)	97(23.4%)	2(0.5%)		
	A little	225(31.8%)	248(35.0%)	226(31.9%)	9(1.3%)		
	Not understand	17(28.8%)	31(52.5%)	10(16.9%)	1(1.7%)		
Q2: Do you understand the application of artificial intelligence in nursing?	Completely	19(50.0%)	6(15.8%)	12(31.6%)	1(2.6%)	22.044	0.006
	Almost	95(40.6%)	86(36.8%)	50(21.4%)	3(1.3%)		
	A little	252(31.3%)	301(37.4%)	244(30.3%)	7(0.9%)		
	Not understand	61(36.5%)	64(38.3%)	40(24.0%)	2(1.2%)		
Q3: What do you think of the current development of artificial intelligence in nursing?	Very good	132(39.5%)	122(36.5%)	76(22.8%)	4(1.2%)	18.943	0.022
	Good	209(32.8%)	231(36.3%)	190(29.8%)	7(1.1%)		
	Average	75(29.6%)	100(39.5%)	77(30.4%)	1(5.3%)		
	Poor	11(57.9%)	4(21.1%)	3(15.8%)	1(5.3%)		
Q4: Do you agree that artificial intelligence will revolutionize the field of nursing?	Strongly agree	108(42.2%)	93(36.3%)	51(19.9%)	4(1.6%)	18.67	0.022
	Agree	242(33.7%)	262(36.4%)	209(29.1%)	6(0.8%)		
	Disagree	66(29.1%)	88(38.8%)	71(31.3%)	2(0.9%)		
	Strongly disagree	11(26.8%)	14(34.1%)	15(36.6%)	1(2.4%)		
Q5: Do you agree that the application of artificial intelligence in nursing can improve patients care?	Strongly agree	155(41.0%)	129(34.1%)	87(23.0%)	7(1.9%)	20.917	0.01
	Agree	248(31.6%)	299(36.8%)	235(28.1%)	4(3.5%)		
	Disagree	18(27.3%)	21(36.8%)	16(28.1%)	2(3.5%)		
	Strongly disagree	6(27.3%)	8(36.4%)	8(36.4%)	0(0.0%)		
Q6: Do you agree that the application of artificial intelligence in nursing can improve nursing decision-making?	Strongly agree	126(41.6%)	108(35.6%)	65(21.5%)	4(1.3%)	15.428	0.069
	Agree	253(32.0%)	291(36.8%)	239(30.3%)	7(0.9%)		
	Disagree	41(31.8%)	52(40.3%)	34(26.4%)	2(1.6%)		
	Strongly disagree	7(33.3%)	6(28.6%)	8(38.1%)	0(0.0%)		
Q7: Do you agree that artificial intelligence in nursing can improve the health of populations?	Strongly agree	124(40.5%)	107(35.0%)	69(22.5%)	6(2.0%)	17.395	0.035
	Agree	267(32.7%)	303(37.1%)	241(29.5%)	5(0.6%)		
	Disagree	28(28.0%)	42(42.0%)	28(28.0%)	2(2.0%)		
	Strongly disagree	8(38.1%)	5(23.8%)	8(38.1%)	0(0.0%)		
Q8: Do you agree that the application of artificial intelligence in nursing will reduce healthcare costs?	Strongly agree	125(39.2%)	120(37.6%)	69(21.6%)	5(1.6%)	10.965	0.256
	Agree	241(33.2%)	262(36.1%)	216(29.8%)	7(1.0%)		
	Disagree	54(31.2%)	65(37.6%)	53(30.6%)	1(0.6%)		
	Strongly disagree	7(28.0%)	10(40.0%)	8(32.0%)	0(0.0%)		
Q9: Do you agree that the application of artificial intelligence will reduce the burden on healthcare workers?	Strongly agree	158(38.4%)	152(37.0%)	95(23.1%)	6(1.5%)	12.644	0.162
	Agree	244(32.3%)	271(35.9%)	233(30.9%)	7(0.9%)		
	Disagree	19(32.8%)	27(46.6%)	12(20.7%)	0(0.0%)		
	Strongly disagree	6(31.6%)	7(36.8%)	6(31.6%)	0(0.0%)		
Q10: Do you agree that artificial intelligence in nursing will change the role of nurses in the future?	Strongly agree	107(42.6%)	85(33.9%)	55(21.9%)	4(1.6%)	16.859	0.041
	Agree	235(32.7%)	273(38.0%)	204(28.4%)	7(1.0%)		
	Disagree	68(29.7%)	87(38.0%)	73(31.9%)	1(0.4%)		
	Strongly disagree	17(38.6%)	12(27.3%)	14(31.8%)	1(2.3%)		
Q11: Do you agree that artificial intelligence in nursing will replace the work of nurses?	Strongly agree	64(41.0%)	54(34.6%)	35(22.4%)	3(1.9%)	8.91	0.446
	Agree	164(32.9%)	180(36.1%)	150(30.1%)	4(0.8%)		
	Disagree	147(34.6%)	162(38.1%)	113(26.6%)	3(0.7%)		
	Strongly disagree	52(31.7%)	61(37.2%)	48(29.3%)	3(1.8%)		
Q12: Do you accept the application of artificial intelligence in nursing?	Strongly agree	157(40.3%)	145(37.2%)	83(21.3%)	5(1.3%)	18.701	0.024
	Agree	260(31.4%)	305(36.8%)	255(30.8%)	8(1.0%)		
	Disagree	8(47.1%)	4(23.5%)	5(29.4%)	0(0.0%)		
	Strongly disagree	2(25.0%)	3(37.5%)	3(37.5%)	0(0.0%)		

**Table 5:** Knowledge and attitudes toward AI in nursing among participants with various categories of professionals.

		Undergraduate nursing students	Postgraduate nursing students	Nurses	Medical professional or technicians (non-nursing)	Artificial intelligence related professionals	Other professionals	Chi-square	*p* value
Q1: Do you understand artificial intelligence?	Completely	10(2.9%)	1(1%)	11(2.9%)	10(6.6%)	24(17.9%)	6(4.4%)	131.856	<0.001
	Almost	109(32.2%)	30(29.4%)	110(29.1%)	50(32.5%)	78(58.2%)	37(27.2%)		
	A little	205(60.5%)	70(68.6%)	228(60.3%)	86(55.8%)	30(22.4%)	89(65.4%)		
	Not understand	15(4.4%)	1(1%)	29(7.7%)	8(5.2%)	2(1.5%)	4(2.9%)		
Q2: Do you understand the application of artificial intelligence in nursing?	Completely	9(2.7%)	1(1%)	6(1.6%)	7(4.5%)	9(6.7%)	6(4.4%)	54.255	<0.001
	Almost	56(16.5%)	17(16.7%)	75(19.8%)	20(13%)	46(34.3%)	20(14.7%)		
	A little	234(69%)	73(71.6%)	252(66.7%)	94(61%)	65(48.5%)	86(63.2%)		
	Not understand	40(11.8%)	11(10.8%)	45(11.9%)	33(21.4%)	14(10.4%)	24(17.6%)		
Q3: What do you think of the current development of artificial intelligence in nursing?	Very good	76(22.4%)	32(31.4%)	90(23.8%)	48(31.2%)	54(40.3%)	34(25%)	24.241	0.061
	Good	187(55.2%)	48(47.1%)	195(51.6%)	73(47.4%)	59(44%)	75(55.1%)		
	Average	72(21.2%)	21(20.6%)	84(22.2%)	31(20.1%)	20(14.9%)	25(18.4%)		
	Poor	4(1.2%)	1(1%)	9(2.4%)	2(1.3%)	1(0.7%)	2(1.5%)		
Q4: Do you agree that artificial intelligence will revolutionize the field of nursing?	Strongly agree	43(12.7%%)	15(14.7%)	86(22.8%)	46(29.9%)	41(30.6%)	25(18.4%)	43.707	<0.001
	Agree	204(60.2%)	60(58.8%)	215(56.9%)	82(53.2%)	79(59%)	79(58.1%)		
	Disagree	79(23.3%)	23(22.5%)	64(16.9%)	23(14.9%)	12(9%)	26(19.1%)		
	Strongly disagree	13(3.8%)	4(3.9%)	13(3.4%)	3(1.9%)	2(1.5%)	6(4.4%)		
Q5: Do you agree that the application of artificial intelligence in nursing can improve patients care?	Strongly agree	77(22.7%)	37(36.3%)	108(28.6%)	54(35.1%)	64(47.8%)	38(27.9%)	40.573	<0.001
	Agree	233(68.7%)	63(61.8%)	242(64%)	93(60.4%)	67(50%)	88(64.7%)		
	Disagree	19(5.6%)	2(2%)	21(5.6%)	4(2.6%)	2(1.5%)	9(6.6%)		
	Strongly disagree	10(2.9%)	0(0%)	7(1.9%)	3(1.9%)	1(0.7%)	1(0.7%)		
Q6: Do you agree that the application of artificial intelligence in nursing can improve nursing decision-making?	Strongly agree	53(15.6%)	23(22.5%)	87(23%)	51(33.1%)	59(44%)	30(22.3%)	57.151	<0.001
	Agree	239(70.5%)	72(70.6%)	240(63.5%)	87(56.7%)	66(49.3%)	86(63.2%)		
	Disagree	38(11.2%)	7(6.9%)	45(11.9%)	13(8.4%)	8(6%)	18(13.2%)		
	Strongly disagree	9(2.7%)	0(0%)	6(1.6%)	3(1.9%)	1(0.7%)	2(1.5%)		
Q7: Do you agree that artificial intelligence in nursing can improve the health of populations?	Strongly agree	54(15.9%)	31(30.4%)	84(22.2%)	52(33.8%)	53(39.6%)	32(23.2%)	58.072	<0.001
	Agree	250(73.7%)	68(66.7%)	248(65.6%)	91(59.1%)	73(54.5%)	86(63.2%)		
	Disagree	24(7.1%)	3(2.9%)	39(10.3%)	9(5.8%)	8(6%)	17(12.5%)		
	Strongly disagree	11(3.3%)	0(0%)	7(1.9%)	2(1.3%)	0(0%)	1(0.7%)		
Q8: Do you agree that the application of artificial intelligence in nursing will reduce healthcare costs?	Strongly agree	50(14.7%)	27(26.5%)	90(23.8%)	60(39%)	58(43.3%)	34(25%)	65.654	<0.001
	Agree	222(65.5%)	61(59.8%)	231(61.1%)	74(48.1%)	63(47%)	75(55.1%)		
	Disagree	57(16.8%)	14(13.7%)	49(13%)	16(10.4%)	12(9%)	25(18.4%)		
	Strongly disagree	10(2.9%)	0(0%)	8(2.1%)	4(2.6%)	1(0.7%)	2(1.5%)		
Q9: Do you agree that the application of artificial intelligence will reduce the burden on healthcare workers?	Strongly agree	83(24.5%)	42(41.2%)	124(32.8%)	56(36.4%)	65(48.5%)	41(30.1%)	41.91	<0.001
	Agree	232(68.4%)	58(56.9%)	231(61.1%)	88(57.1%)	63(61%)	83(61%)		
	Disagree	21(6.2%)	2(2%)	13(3.4%)	6(3.9%)	6(4.5%)	10(7.4%)		
	Strongly disagree	3(0.9%)	0(0%)	10(2.6%)	4(2.6%)	0(0%)	2(1.5%)		
Q10: Do you agree that artificial intelligence in nursing will change the role of nurses in the future?	Strongly agree	45(13.3%)	22(21.6%)	73(19.3%)	42(27.3%)	39(29.1%)	30(22.1%)	43.953	<0.001
	Agree	200(59%)	53(52%)	240(63.5%)	87(56.5%)	67(50%)	72(52.9%)		
	Disagree	82(24.2%)	23(22.5%)	55(14.6%)	16(10.4%)	26(19.4%)	27(19.9%)		
	Strongly disagree	12(3.5%)	4(3.9%)	10(2.6%)	9(5.8%)	2(1.5%)	7(5.1%)		
Q11: Do you agree that artificial intelligence in nursing will replace the work of nurses?	Strongly agree	28(8.3%)	9(8.8%)	46(12.2%)	30(19.5%)	26(19.4%)	17(12.5%)	43.628	<0.001
	Agree	127(37.5%)	34(33.3%)	170(45%)	62(40.3%)	51(38.1%)	54(39.7%)		
	Disagree	132(38.9%)	34(33.3%)	130(34.4%)	43(27.9%)	40(29.9%)	46(33.8%)		
	Strongly disagree	52(15.3%)	25(24.5%)	32(8.5%)	19(12.3%)	17(12.7%)	19(14%)		
Q12: Do you accept the application of artificial intelligence in nursing?	Strongly agree	76(22.4%)	41(40.2%)	129(34.1%)	52(33.8%)	55(41%)	37(27.2%)	32.8	<0.001
	Agree	256(75.5%)	61(59.8%)	241(63.8%)	99(64.3%)	77(57.5%)	94(69.1%)		
	Disagree	4(1.2%)	0(0%)	6(1.6%)	1(0.6%)	1(0.7%)	5(3.7%)		
	Strongly disagree	3(0.9%)	0(0%)	2(0.5%)	2(1.3%)	1(0.7%)	0(0%)		

**Table 6:** Knowledge and attitudes toward AI in nursing across participants with varying educational backgrounds.

		College or less	Bachelor’s degree (or bachelor’s degree in progress)	Master’s degree (or master’s degree in progress)	Doctoral degree or higher (or doctoral degree in progress)	Chi-square	*p* value
Q1: Do you understand artificial intelligence?	Completely	15(24.2%)	27(43.5%)	8(12.9%)	12(19.4%)	70.598	<0.001
	Almost	66(15.9%)	237(57.2%)	86(20.8%)	25(6.0%)		
	A little	158(22.3%)	418(59.0%)	119(16.8%)	13(1.8%)		
	Not understand	19(32.2%)	36(61.0%)	4(6.8%)	0(0.0%)		
Q2: Do you understand the application of artificial intelligence in nursing?	Completely	11(28.9%)	21(55.3%)	3(7.9%)	3(7.9%)	22.053	0.009
	Almost	52(22.2%)	121(51.7%)	49(20.9%)	12(5.1%)		
	A little	164(20.4%)	483(60.1%)	136(16.9%)	21(2.6%)		
	Not understand	31(18.6%)	93(55.7%)	29(17.4%)	14(8.4%)		
Q3: What do you think of the current development of artificial intelligence in nursing?	Very good	62(18.6%)	176(52.7%)	74(22.2%)	22(6.6%)	23.193	0.004
	Good	143(22.4%)	380(59.7%)	100(15.7%)	14(2.2%)		
	Average	49(19.4%)	152(60.1%)	40(15.8%)	12(4.7%)		
	Poor	4(21.1%)	10(52.6%)	3(15.8%)	2(10.5%)		
Q4: Do you agree that artificial intelligence will revolutionize the field of nursing?	Strongly agree	55(21.5%)	130(50.8%)	51(19.9%)	20(7.8%)	16.711	0.053
	Agree	147(20.4%)	423(58.8%)	126(17.5%)	23(3.2%)		
	Disagree	48(21.1%)	139(61.2%)	34(15.0%)	6(2.6%)		
	Strongly disagree	8(19.5%)	26(63.4%)	6(14.6%)	1(2.4%)		
Q5: Do you agree that the application of artificial intelligence in nursing can improve patients care?	Strongly agree	66(17.5%)	200(52.9%)	83(22.0%)	29(7.7%)	32.164	<0.001
	Agree	174(22.1%)	463(58.9%)	128(16.3%)	21(2.7%)		
	Disagree	13(22.8%)	39(68.4%)	5(8.8%)	0(0.0%)		
	Strongly disagree	5(22.7%)	16(72.7%)	1(4.5%)	0(0.0%)		
Q6: Do you agree that the application of artificial intelligence in nursing can improve nursing decision-making?	Strongly agree	61(20.1%)	151(49.8%)	65(21.5%)	26(8.6%)	33.235	<0.001
	Agree	169(21.4%)	462(58.5%)	138(17.5%)	21(2.7%)		
	Disagree	24(18.6%)	90(69.8%)	12(9.3%)	3(2.3%)		
	Strongly disagree	4(19.0%)	15(71.4%)	2(9.5%)	0(0.0%)		
Q7: Do you agree that artificial intelligence in nursing can improve the health of populations?	Strongly agree	59(19.3%)	149(48.7%)	73(23.9%)	25(8.2%)	32.683	<0.001
	Agree	171(21.0%)	491(60.2%)	131(16.1%)	23(2.8%)		
	Disagree	23(23.0%)	63(63.0%)	12(12.0%)	2(2.0%)		
	Strongly disagree	5(23.8%)	15(71.4%)	1(4.8%)	0(0.0%)		
Q8: Do you agree that the application of artificial intelligence in nursing will reduce healthcare costs?	Strongly agree	64(20.1%)	151(47.3%)	74(23.2%)	30(9.4%)	48.134	<0.001
	Agree	150(20.7%)	448(61.7%)	113(15.6%)	15(2.1%)		
	Disagree	39(22.5%)	103(59.5%)	26(15.0%)	5(2.9%)		
	Strongly disagree	5(20.0%)	16(64.0%)	4(16.0%)	0(0.0%)		
Q9: Do you agree that the application of artificial intelligence will reduce the burden on healthcare workers?	Strongly agree	75(18.2%)	211(51.3%)	94(22.9%)	31(7.5%)	35.37	<0.001
	Agree	169(22.4%)	455(60.3%)	113(15.0%)	18(2.4%)		
	Disagree	9(15.5%)	39(67.2%)	9(15.5%)	1(1.7%)		
	Strongly disagree	5(26.3%)	13(68.4%)	1(5.3%)	0(0.0%)		
Q10: Do you agree that artificial intelligence in nursing will change the role of nurses in the future?	Strongly agree	49(19.5%)	130(51.8%)	54(21.5%)	18(7.2%)	15.478	0.079
	Agree	157(21.8%)	424(59.0%)	116(16.1%)	22(3.1%)		
	Disagree	43(18.8%)	139(60.7%)	40(17.5%)	7(3.1%)		
	Strongly disagree	9(20.5%)	25(56.8%)	7(15.9%)	3(6.8%)		
Q11: Do you agree that artificial intelligence in nursing will replace the work of nurses?	Strongly agree	39(25.0%)	76(48.7%)	30(19.2%)	11(7.1%)	33.923	<0.001
	Agree	122(24.5%)	286(57.6%)	78(15.7%)	12(2.4%)		
	Disagree	75(17.6%)	267(62.8%)	68(16.0%)	15(3.5%)		
	Strongly disagree	22(13.4%)	89(54.3%)	41(25.0%)	12(7.3%)		
Q12: Do you accept the application of artificial intelligence in nursing?	Strongly agree	70(17.9%)	203(52.1%)	92(23.6%)	25(6.4%)	28.117	<0.001
	Agree	181(21.9%)	500(60.4%)	123(14.9%)	24(2.9%)		
	Disagree	5(29.4%)	10(58.8%)	2(11.8%)	0(0.0%)		
	Strongly disagree	2(25.0%)	5(62.5%)	0(0.0%)	1(12.5%)		

**Table 7:** Knowledge and attitudes toward AI in nursing among nurses of various professional titles.

		Junior	Intermediate	Senior	Chi-square	*p* value
Q1: Do you understand artificial intelligence?	Completely or almost	56(46.3%)	51(42.1%)	14(11.6%)	1.006	0.605
	A little or not understand	127(49.4%)	95(37%)	35(13.6%)		
Q2: Do you understand the application of artificial intelligence in nursing?	Completely or almost	41(50.6%)	32(39.5%)	8(9.9%)	0.882	0.643
	A little or not understand	142(47.8%)	114(38.4%)	41(13.8%)		
Q3: What do you think of the current development of artificial intelligence in nursing?	Very good or good	132(46.3%)	111(38.9%)	42(14.7%)	3.895	0.143
	Average or poor	51(54.8%)	35(37.6%)	7(7.5%)		
Q4: Do you agree that artificial intelligence will revolutionize the field of nursing?	Strongly agree or agree	141(46.8%)	120(39.9%)	40(13.3%)	1.463	0.481
	Disagree or strongly disagree	42(54.5%)	26(33.8%)	9(11.7%)		
Q5: Do you agree that the application of artificial intelligence in nursing can improve patients care?	Strongly agree or agree	169(48.3%)	135(38.6%)	46(13.1%)	0.137	0.934
	Disagree or strongly disagree	14(50.0%)	11(39.3)	3(10.7%)		
Q6: Do you agree that the application of artificial intelligence in nursing can improve nursing decision-making?	Strongly agree or agree	156(47.7%)	128(39.1%)	43(13.1%)	0.484	0.785
	Disagree or strongly disagree	27(52.9%)	18(35.3%)	6(11.8%)		
Q7: Do you agree that artificial intelligence in nursing can improve the health of populations?	Strongly agree or agree	167(50.3%)	125(37.7%)	40(12.0%)	4.441	0.109
	Disagree or strongly disagree	16(34.8%)	21(45.7%)	9(19.6%)		
Q8: Do you agree that the application of artificial intelligence in nursing will reduce healthcare costs?	Strongly agree or agree	156(48.6%)	123(38.3%)	42(13.1%)	0.091	0.956
	Disagree or strongly disagree	27(47.4%)	23(40.4%)	7(12.3%)		
Q9: Do you agree that the application of artificial intelligence will reduce the burden on healthcare workers?	Strongly agree or agree	174(49.0%)	138(38.9%)	43(12.1%)	3.783	0.151
	Disagree or strongly disagree	9(39.1%)	8(34.8%)	6(26.1%)		
Q10: Do you agree that artificial intelligence in nursing will change the role of nurses in the future?	Strongly agree or agree	145(46.3%)	127(40.6%)	41(13.1%)	3.457	0.178
	Disagree or strongly disagree	38(58.5%)	19(29.2%)	8(12.3%)		
Q11: Do you agree that artificial intelligence in nursing will replace the work of nurses?	Strongly agree or agree	107(49.5%)	85(39.4%)	24(11.1%)	1.534	0.464
	Disagree or strongly disagree	76(46.9%)	61(37.7%)	25(15.4%)		
Q12: Do you accept the application of artificial intelligence in nursing?	Strongly agree or agree	177(47.8%)	144(38.9%)	49(13.2%)	1.804	0.360
	Disagree or strongly disagree	6(75.0%)	2(25.0%)	0(0.0%)		

### 3.3 Attitudes toward AI

**Figure 3:**
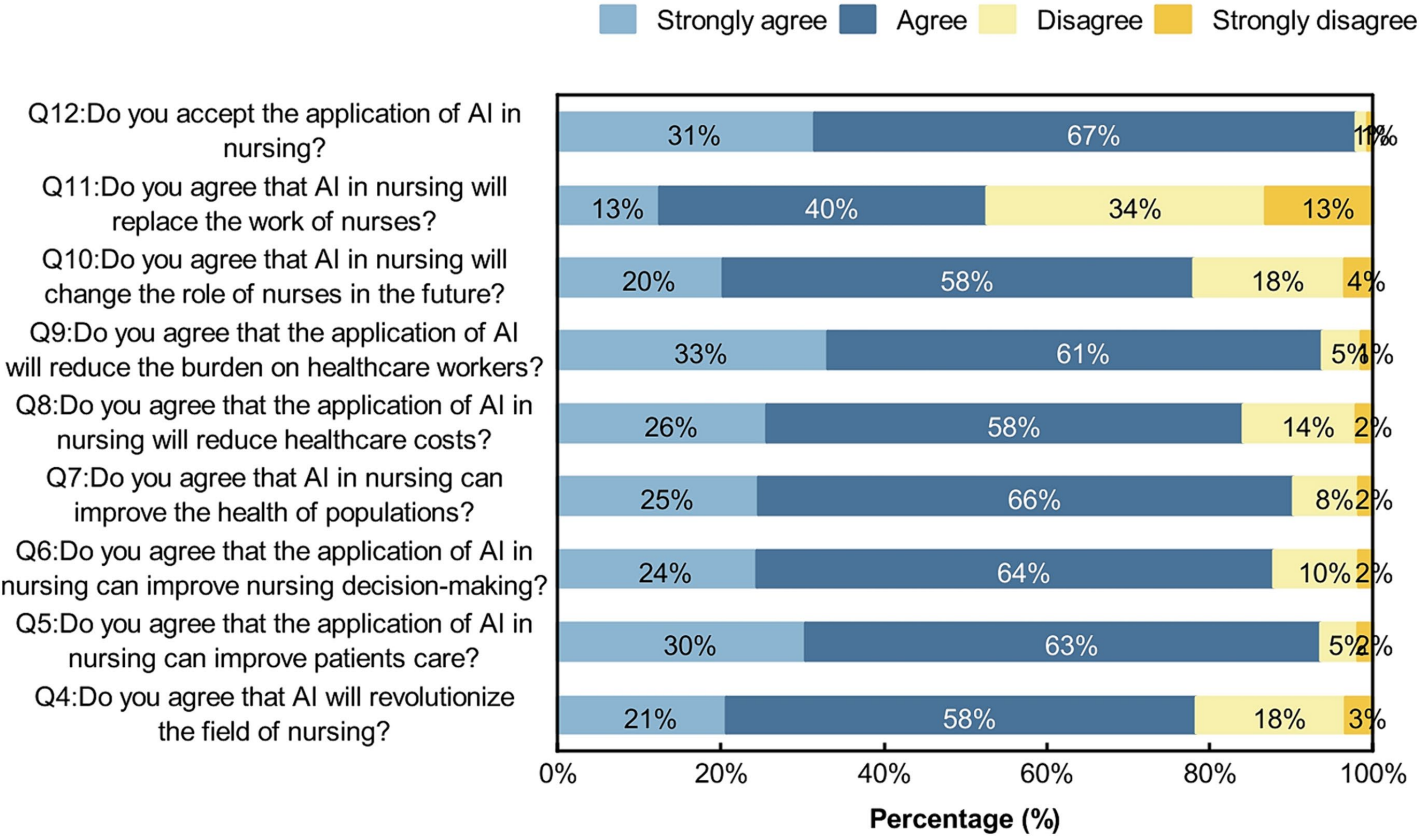
Participants’ attitudes toward AI in nursing.

**Figure 4:**
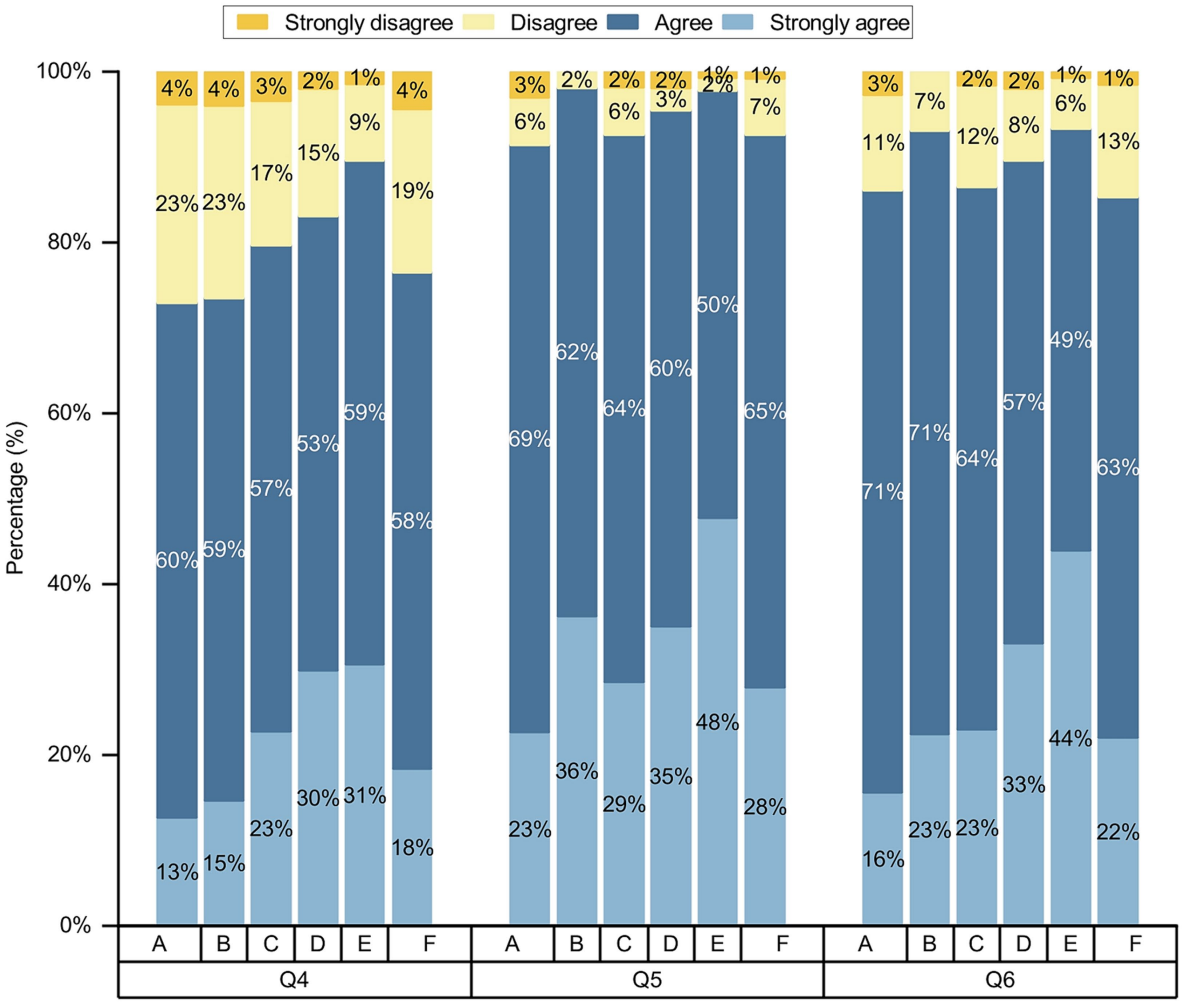
Comparison of differences in attitudes toward AI in nursing among participants with various categories of professionals. **(A)** Undergraduate nursing students; **(B)** postgraduate nursing students; **(C)** nurses; **(D)** other healthcare professionals; **(E)** AI-related professionals; and **(F)** other professionals; Q4–Q6, the questions of attitudes toward AI in nursing.

**Figure 5:**
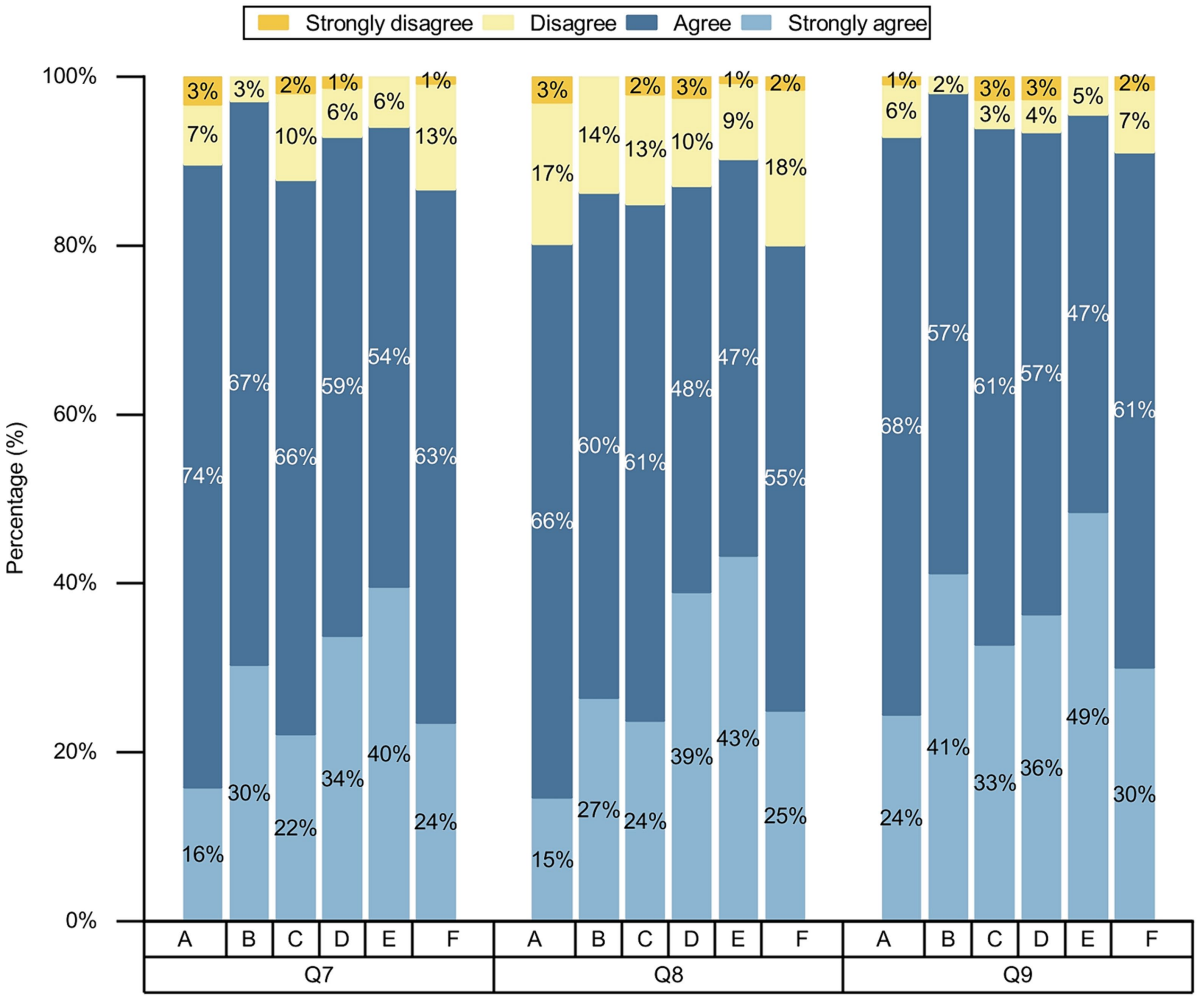
Comparison of differences in attitudes toward AI in nursing among participants with various categories of professionals. **(A)** Undergraduate nursing students; **(B)** postgraduate nursing students; **(C)** nurses; **(D)** other healthcare professionals; **(E)** AI-related professionals; and **(F)** other professionals; Q7–Q9, the questions of attitudes toward AI in nursing.

**Figure 6:**
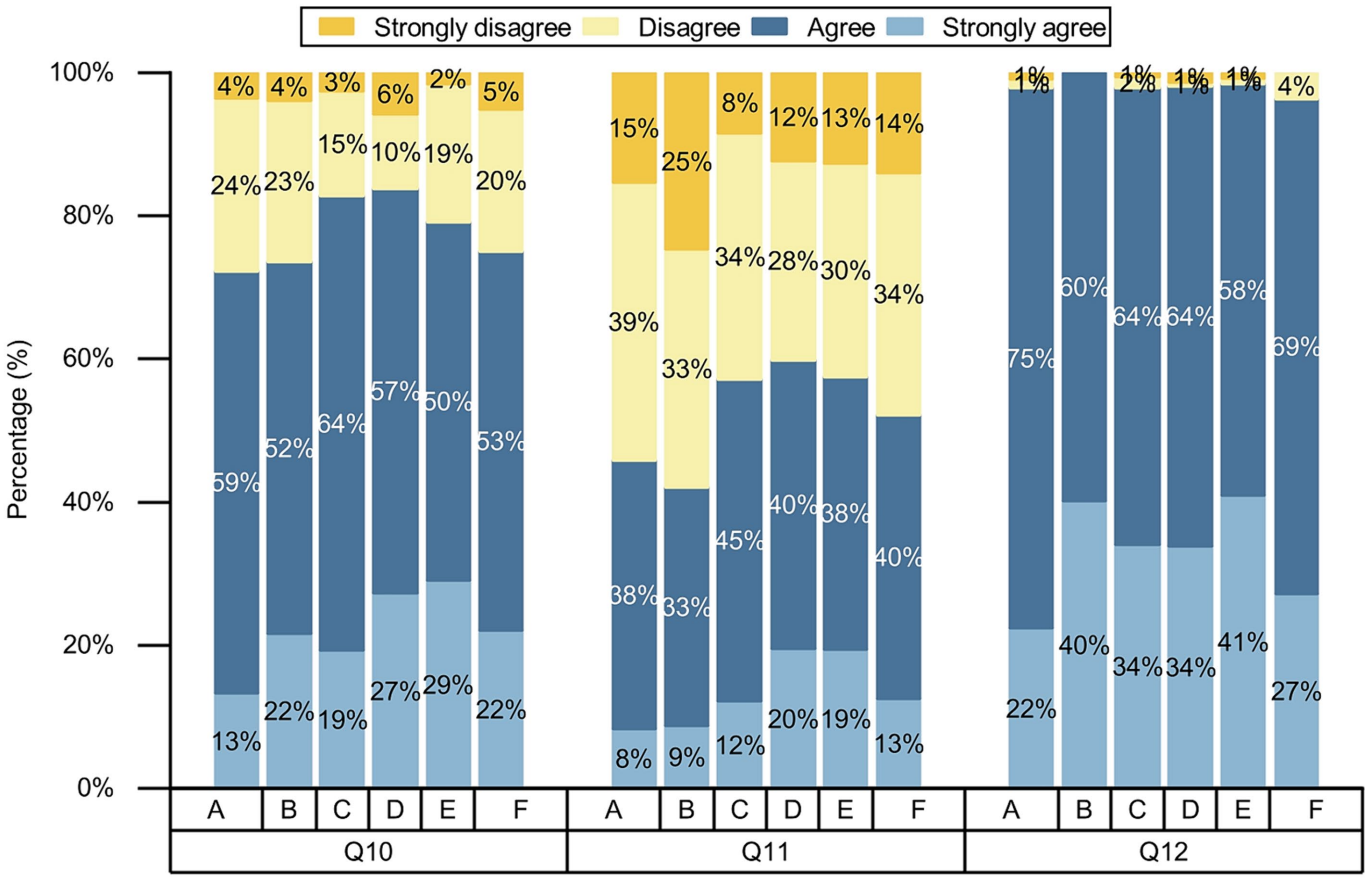
Comparison of differences in attitudes toward AI in nursing among participants with various categories of professionals. **(A)** Undergraduate nursing students; **(B)** postgraduate nursing students; **(C)** nurses; **(D)** other healthcare professionals; **(E)** AI-related professionals; and **(F)** other professionals; Q10–Q12, the questions of attitudes toward AI in nursing.

Participants were positive about AI in nursing, with more than 50% agreeing and strongly agreeing with each question (Figure 3). We conducted chi-square test for gender, age, place of residence, various categories of professionals, educational backgrounds, and professional title level, and the results showed that the *p* values of different genders, ages, and various categories of professionals on the attitudes toward nursing AI were less than 0.05, while the *p* values of different place of residences and varying educational backgrounds on attitudes toward nursing AI part of the question were less than 0.05, as shown on Tables 2–6 and Figures 4–6. *p* values of nurses with different professional title level on attitudes toward nursing AI were all greater than 0.05, as shown on Table 7.

### 3.4 Concerns and ethical research about AI

**Figure 7:**
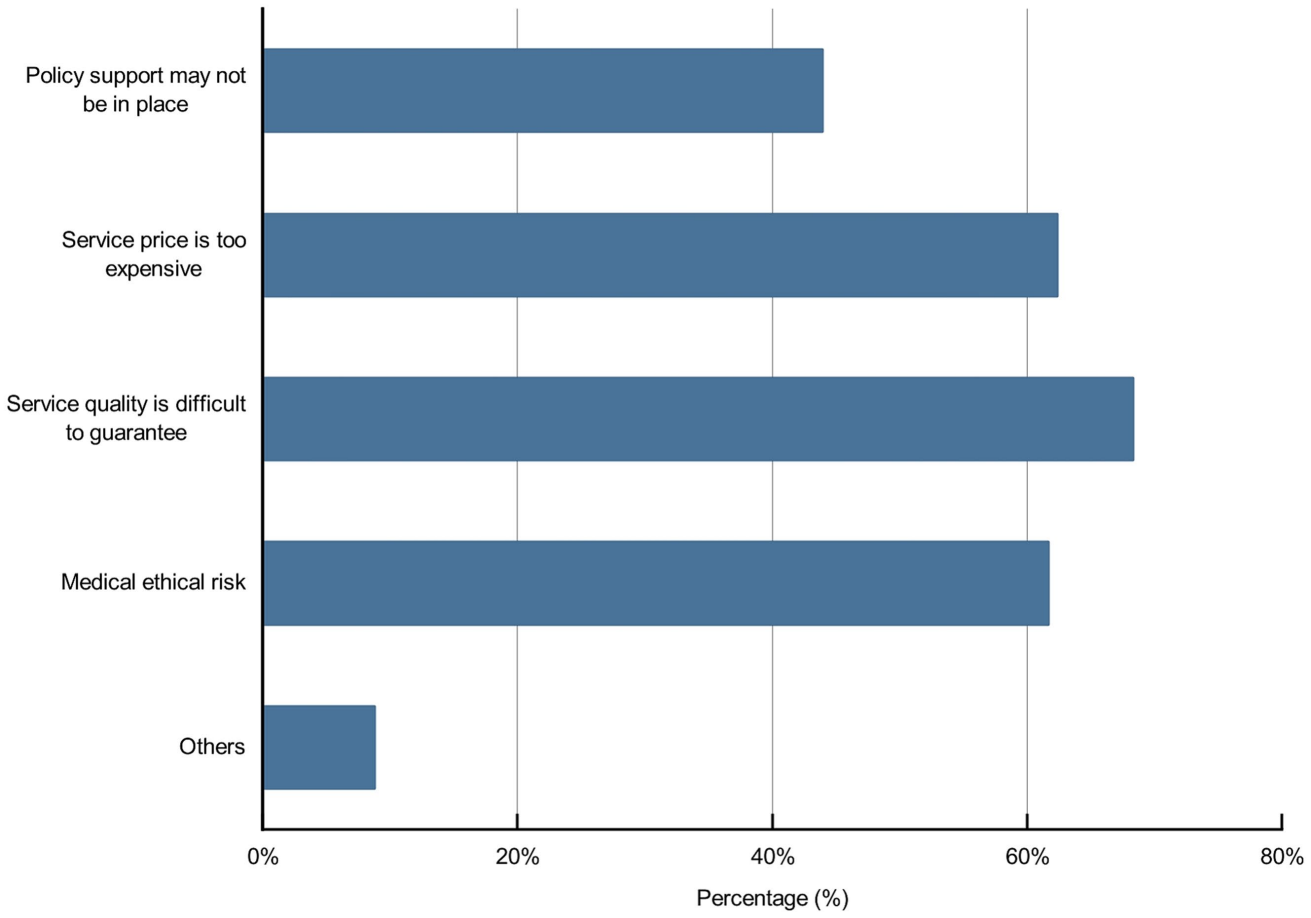
Participants’ concerns toward AI in nursing.

**Figure 8:**
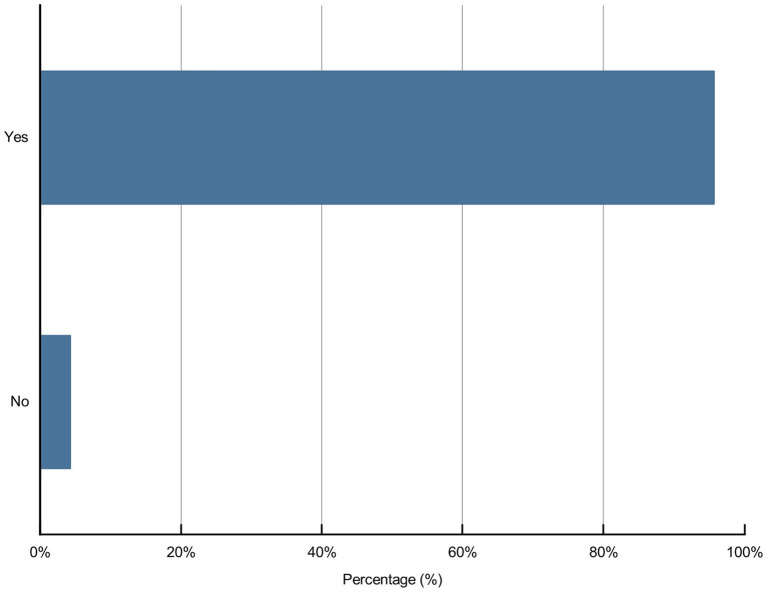
Responses to the necessity for medical ethics toward AI in nursing among participants.

**Figure 9:**
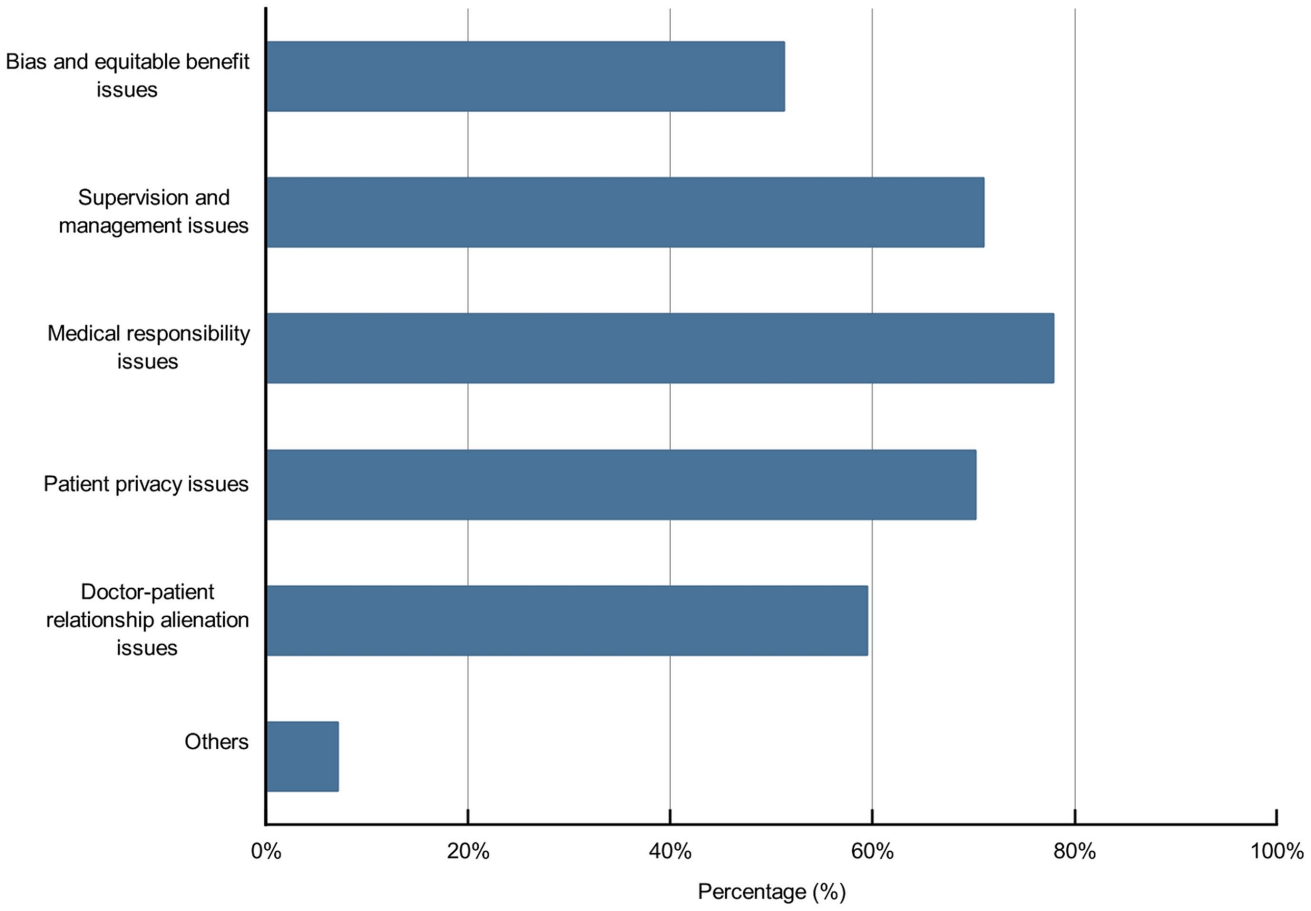
Medical ethical issues toward AI in nursing waiting to be addressed.

Artificial intelligence in nursing may raise some concerns; 43.9, 62.3, 68.3, 61.7, and 8.8% of the participants, respectively, believed that policy support might not be in place, service price was too high, service quality was difficult to guarantee, medical ethical risk and others may hinder the development of AI in nursing (Figure 7). Moreover, 95.7% of participants believed that it was necessary to strengthen medical ethics toward AI in nursing; among these participants, 51.2, 71, 77.8, 70.2, 59.5, and 7.1% of the participants, respectively, believed that they should deal with bias and equitable benefit issues, supervision and management issues, medical responsibility issues, patient privacy issues, nurse–patient relationship alienation issues, and other issues (Figures 8, 9).

## 4 Discussion

This study explored the knowledge, attitudes, and concerns of undergraduate and postgraduate nursing students, nurses, other healthcare professionals, AI-related professionals, and others in China toward AI in nursing. It demonstrated that only a small percentage (38.3 vs. 21.9%) of the 1,243 participants completely or almost completely understood AI and AI in nursing. Most participants had positive attitudes toward AI in nursing. All participants reported concerns about AI in nursing, and 95.7% of the participants showed that it was necessary to perform ethical research on AI in nursing.

Our study shows that there are differences on knowledge of AI and AI in nursing by gender, age, place of residence, various categories of professionals, and educational backgrounds, and that there are differences on attitudes toward nursing AI by gender, age, and various categories of professionals, which is similar to the findings of Syed et al. (37) and Kansal et al. (38). However, similar study from Bahrain reported no correlations between the responses obtained and the age, gender or year of professional experience of the participants (29). This may be related to the high internet penetration in Bahrain and the younger age of the participants.

### 4.1 Knowledge of AI and AI in nursing

This study revealed that the participants had a low level of knowledge of AI and lacked knowledge of AI, particularly in nursing. Moreover, undergraduate nursing students, postgraduate nursing students, nurses, and other healthcare professionals significantly lacked knowledge of AI or AI in nursing compared with AI-related professionals. Our study showed that only 35.1% of undergraduate nursing students and 30.4% of postgraduate nursing students reported a complete or almost complete understanding of AI, and only 19.3% of undergraduate nursing students and 17.7% of graduate nursing students reported a complete or almost complete understanding of f AI’s application in nursing. This observation is consistent with previous studies that medical students in developing and developed countries have moderate or low levels of AI knowledge (32, 39–41). Significant differences were observed in the findings of Doumat et al. (42), Swed et al. (43), and Stewart (31), who showed that 59.7% of Lebanese medical students, 70% of Syrian medical students, and 84.8% of Western Australian medical students showed a good knowledge of AI and had a good understanding or possessed basic knowledge of AI, respectively. However, it was also revealed that most medical students reported little understanding of the basic computational principles of AI and AI’s application in medicine in their studies (31, 43). This study showed that only 32% of nurses and 39.1% of other healthcare professionals were completely or almost understood AI, and only 21.4% of nurses and 17.5% of healthcare professionals were completely or almost completely understood AI’s application in nursing, consistent with previous studies (44). In response to the general lack of awareness of AI among medical students and healthcare professionals, the level of awareness can be increased by incorporating AI into medical school curricula or by increasing AI-related training (45, 46).

### 4.2 Attitudes toward AI in nursing

Our study shows that there is little difference in the attitudes of different professionals and technicians toward the use of AI in nursing, which is generally optimistic. Most participants believed that using AI in nursing could improve patient care, population health, and nursing decisions and reduce the burden on healthcare professionals. Positive attitudes of nursing students and nurses toward AI are conducive to the development of AI in nursing. The more positive the attitude toward AI, the more it will help nursing students use AI technology in their future work and overcome potential barriers to AI technology (47). However, 53% of participants agreed or strongly agreed that AI would replace the jobs of nurses, inconsistent with the study by Moldt et al. (28), where most medical students did not believe that AI would replace their jobs. The survey by Castagno et al. (44) of Saudi Arabian healthcare workers also found that 72% of participants were not concerned that AI would replace their jobs. The reason for this discrepancy may be that the participants in this study lacked knowledge about AI and did not realize that AI technology has emerged to complement the work of healthcare workers and enhance their productivity rather than replace the work of nurses.

### 4.3 Nurses’ knowledge and attitudes toward AI in nursing

This study showed no differences in knowledge and attitudes toward AI in nursing among nurses with different job titles and generally less understanding of AI but positive attitudes. The lack of nurses’ understanding of AI may be due to several reasons. First, AI’s development in China’s nursing field is in its initial stage, and the application of AI technology in nursing is not yet well understood. Second, knowledge of AI technology often involves complex computer disciplines, and some AI algorithms exhibit the “black box” phenomenon, making AI technology difficult to understand (48). Third, due to the shortage of nurses in China, Chinese nurses working in hospitals are often physically and mentally exhausted because of their busy schedules and lack of time to learn AI (49). However, nurses had a positive attitude toward AI, which benefits the dissemination of AI-related knowledge and the application of AI technology.

In addition, healthcare professionals, especially nurses, are the main contributors to the promotion of AI in nursing, and their attitudes toward AI in nursing will, to a certain extent, affect the speed and quality of nursing AI development. However, our study showed no difference between nurses and other professionals in their attitudes toward AI in nursing and do not show significant advantages, so the popularization of AI knowledge among nurses should be strengthened to enhance the promotion of AI in the nursing field.

### 4.4 Concerns about AI in nursing

Although most participants were positive about the application of AI in nursing, all expressed concerns. Difficulty guaranteeing the quality of AI, being too expensive to use, many medical ethical issues, and the lack of policy support were the top four reasons hindering the application of AI in the nursing field. In China, AI technology is slowly integrating into nursing disciplines, and we need to fully understand the strengths and limitations of AI and address or improve these obstacles in practice to promote the development of the nursing field better.

### 4.5 Ethical issues

This study showed that 95.7% of the participants thought it necessary to strengthen medical ethics toward AI in nursing, where the first issue of attribution of medical responsibility was of great concern. Who is responsible for the medical errors when healthcare professionals use AI technology? However, this issue is controversial (50, 51). The second issue concerns the supervision and management of AI technology. In China, a supervision, management system and guidelines for AI have not been established, which may lead to the misuse of AI technology and loss of management control by healthcare administrators (24). Further, ethical issues regarding patient privacy and safety exist (52). AI technology requires a large amount of patient data, and the use of patient health-related data may involve personal information, resulting in patient privacy issues (24). In addition, using AI technology may lead to alienation of the nurse–patient relationship. Nursing is a discipline of humanistic care, and AI technology cannot communicate well with patients emotionally (53).

Regarding these ethical issues, healthcare professionals need to approach AI technology dialectically. They cannot do so without managers, research developers, and healthcare professionals continuing to improve AI technology or develop AI-related legal regimes to facilitate the development of AI in healthcare.

### 4.6 Strengths and limitations

This study had several strengths. First, to develop the questionnaire, we reviewed the relevant literature, conducted expert consultations, and performed a pilot study before starting the study to ensure its reliability and validity. This ensured the rigor of the study. Second, to make a good comparison, we surveyed six groups of people, compared their knowledge and attitudes toward AI in nursing, and analyzed and compared the differences. Third, few studies have investigated the ethical issues of AI; in contrast, this study investigated and discussed participants’ perceptions of ethical research on AI. Finally, this is the first study conducted in China on knowledge and attitudes toward AI in nursing, which lays the foundation for the development of AI in nursing.

This study also had limitations. First, we used the snowball sampling method, which might lead to information bias, affecting the generalization of the study’s findings. Second, this was a cross-sectional study and did not analyze the relationships between variables in depth. Further in-depth studies are required in the future.

## 5 Conclusion

The application of AI in healthcare is an important public health issue. This study showed that nursing students and healthcare professionals lacked knowledge about AI and its application in nursing but had a positive attitude toward AI overall. Due to this lack of knowledge, there is a need to include AI in medical students’ curricula or to add relevant training. The majority of participants believed that AI would replace nurses’ jobs, which might be related to their insufficient understanding of AI. Every participant expressed concerns about nursing AI, and the vast majority agreed on the need for medical ethics research on nursing AI. With the continuous development of AI in healthcare, it is essential for healthcare professionals to deepen their understanding of AI, accept it with an open and positive attitude, and use AI technology reasonably to solve clinical problems.

The study did not find that clinical nursing professionals had an advantage over non-clinical nursing personnel in terms of their knowledge, attitudes, and concerns about nursing AI. It is important to highlight that clinical nursing personnel are the primary drivers of research and practice in nursing AI. Their knowledge, attitudes, and concerns significantly impact the development and application of nursing AI. As challenges and ethical issues exist in using AI technology, healthcare professionals must approach AI technology dialectically. They cannot work independently of managers, researchers, developers, and other healthcare professionals if they are to continuously improve AI technology and develop AI-related legal systems to promote AI advancement in healthcare. The findings of this study could provide valuable insights for developing new strategies to enhance healthcare services.

## Data availability statement

The raw data supporting the conclusions of this article will be made available by the authors, without undue reservation.

## Author contributions

XW: Data curation, Formal analysis, Methodology, Writing – original draft. FF: Investigation, Writing – original draft, Conceptualization, Methodology. JW: Data curation, Investigation, Writing – original draft. MH: Formal analysis, Methodology, Writing – original draft. FX: Formal analysis, Investigation, Writing – original draft. JT: Formal analysis, Writing – original draft. YW: Project administration, Supervision, Writing – review & editing. JG: Project administration, Resources, Supervision, Writing – review & editing.

## References

[1] Ah S Ha S. Artificial intelligence education and tools for medical and health informatics students: systematic review. JMIR Med Educ. (2020) 6:e19285. doi: 10.2196/19285 32602844 PMC7367541

[2] O'Connor S Gasteiger N Stanmore E Wong DC Lee JJ. Artificial intelligence for falls management in older adult care: a scoping review of nurses’ role. J Nurs Manag. (2022) 30:3787–801. doi: 10.1111/jonm.13853 36197748 PMC10092211

[3] Kulkarni S Seneviratne N Baig MS Khan AHA. Artificial intelligence in medicine: where are we now? Acad Radiol. (2020) 27:62–70. doi: 10.1016/j.acra.2019.10.001 31636002

[4] Stewart J Sprivulis P Dwivedi G. Artificial intelligence and machine learning in emergency medicine. Emerg Med Austral. (2018) 30:870–4. doi: 10.1111/1742-6723.13145 30014578

[5] Al F Jc K. Artificial intelligence powers digital medicine. NPJ Digit Med. (2018) 1:5. doi: 10.1038/s41746-017-0012-2 31304291 PMC6548340

[6] Cheng JZ Ni D Chou YH Qin J Tiu CM Chang YC. Computer-aided diagnosis with deep learning architecture: applications to breast lesions in US images and pulmonary nodules in CT scans. Sci Rep. (2016) 6:24454. doi: 10.1038/srep24454 27079888 PMC4832199

[7] Vaughn J Shaw RJ Molloy MA. A telehealth case study: the use of telepresence robot for delivering integrated clinical care. J Am Psychiatr Nurses Assoc. (2015) 21:431–2. doi: 10.1177/1078390315617037 26597906

[8] Ma AC Meng Z Ding X. Performance review of intelligent guidance robot at the outpatient clinic setting. Cureus. (2021) 13:e16840. doi: 10.7759/cureus.16840 34522486 PMC8425029

[9] Li Y Wang M Wang L Cao Y Liu Y Zhao Y. Advances in the application of AI robots in critical care: scoping review. J Med Internet Res. (2024) 26:e54095. doi: 10.2196/54095 38801765 PMC11165292

[10] Yoo HJ Kim EH Lee H. Mobile robots for isolation-room hospital settings: a scenario-based preliminary study. Comput Struct Biotechnol J. (2024) 24:237–46. doi: 10.1016/j.csbj.2024.03.001 38572167 PMC10990676

[11] Sun Y Xiao C Chen L Chen L Lu H Wang Y. A review of intelligent walking support robots: aiding Sit-to-stand transition and walking. IEEE Trans Neural Syst Rehabil Eng. (2024) 32:1355–69. doi: 10.1109/TNSRE.2024.3379453 38502616

[12] Zhao D Sun X Shan B Yang Z Yang J Liu H. Research status of elderly-care robots and safe human-robot interaction methods. Front Neurosci. (2023) 17:1291682. doi: 10.3389/fnins.2023.1291682 38099199 PMC10720664

[13] Yang J Gong Y Yu L Peng L Cui Y Huang H. Effect of exoskeleton robot-assisted training on gait function in chronic stroke survivors: a systematic review of randomised controlled trials. BMJ Open. (2023) 13:e074481. doi: 10.1136/bmjopen-2023-074481 PMC10503387 37709309

[14] Nichol B McCready J Erfani G Comparcini D Simonetti V Cicolini G. Exploring the impact of socially assistive robots on health and wellbeing across the lifespan: an umbrella review and meta-analysis. Int J Nurs Stud. (2024) 153:104730. doi: 10.1016/j.ijnurstu.2024.104730 38430662

[15] Ni Z Peng R Zheng X Xie P. Embracing the future: integrating ChatGPT into China’s nursing education system. Int J Nurs Sci. (2024) 11:295–9. doi: 10.1016/j.ijnss.2024.03.006 38707690 PMC11064564

[16] Abujaber AA Abd-alrazaq A al-Qudimat AR Nashwan AJ. A strengths, weaknesses, opportunities, and threats (SWOT) analysis of ChatGPT integration in nursing education: a narrative review. Cureus. (2023) 15:e48643. doi: 10.7759/cureus.48643 38090452 PMC10711690

[17] Mk C My K. Enhancing nursing competency through virtual reality simulation among nursing students: a systematic review and meta-analysis. Front Med. (2024). 11:1351300. doi: 10.3389/fmed.2024.1351300 PMC11106392 38774395

[18] Berşe S Akça K Dirgar E Kaplan Serin E. The role and potential contributions of the artificial intelligence language model ChatGPT. Ann Biomed Eng. (2024) 52:130–3. doi: 10.1007/s10439-023-03296-w 37378876

[19] Alexandre C Pamela F Shauna D Laura V Manal K Tracie R. Revolutionizing nursing education through Ai integration: A reflection on the disruptive impact of ChatGPT. Nurse Educ Today. (2023) 129:105916. doi: 10.1016/j.nedt.2023.105916 37515957

[20] D’Ambrosio F Harbo M Contiero D Bonfigli AR Cicconi D Heuer N. Preact to lower the risk of falling by customized rehabilitation across Europe: the feasibility study protocol of the PRECISE project in Italy. Front Public Health. (2024) 12:1293621. doi: 10.3389/fpubh.2024.1293621 38584921 PMC10996439

[21] Padula WV Armstrong DG Pronovost PJ Saria S. Predicting pressure injury risk in hospitalised patients using machine learning with electronic health records: a US multilevel cohort study. BMJ Open. (2024) 14:e082540. doi: 10.1136/bmjopen-2023-082540 PMC11146395 38594078

[22] Digumarthi V Amin T Kanu S Mathew J Edwards B Peterson LA. Preoperative prediction model for risk of readmission after total joint replacement surgery: a random forest approach leveraging NLP and unfairness mitigation for improved patient care and cost-effectiveness. J Orthop Surg. (2024) 19:287. doi: 10.1186/s13018-024-04774-0 PMC11084055 38725085

[23] Gong D Chen X Yang L Zhang Y Zhong Q Liu J. From normal population to prediabetes and diabetes: study of influencing factors and prediction models. Front Endocrinol. (2023) 14:1225696. doi: 10.3389/fendo.2023.1225696 PMC10640999 37964953

[24] Lee D Yoon SN. Application of artificial intelligence-based Technologies in the Healthcare Industry: opportunities and challenges. Int J Environ Res Public Health. (2021) 18:271. doi: 10.3390/ijerph18010271 33401373 PMC7795119

[25] Abdullah R Fakieh B. Health care employees’ perceptions of the use of artificial intelligence applications: survey study. J Med Internet Res. (2020) 22:e17620. doi: 10.2196/17620 32406857 PMC7256754

[26] Davoudi A Malhotra KR Shickel B Siegel S Williams S Ruppert M. Intelligent ICU for autonomous patient monitoring using pervasive sensing and deep learning. Sci Rep. (2019) 9:8020. doi: 10.1038/s41598-019-44004-w 31142754 PMC6541714

[27] Fritsch SJ Blankenheim A Wahl A Hetfeld P Maassen O Deffge S. Attitudes and perception of artificial intelligence in healthcare: a cross-sectional survey among patients. Digit Health. (2022) 8:205520762211167. doi: 10.1177/20552076221116772 PMC9380417 35983102

[28] Moldt J-A Festl-Wietek T Madany Mamlouk A Nieselt K Fuhl W Herrmann-Werner A. Chatbots for future docs: exploring medical students’ attitudes and knowledge towards artificial intelligence and medical chatbots. Med Educ. (2023) 28:2182659. doi: 10.1080/10872981.2023.2182659 PMC9979998 36855245

[29] Al-Medfa MK Al-Ansari AMS Darwish AH Qreeballa TA Jahrami H. Physicians’ attitudes and knowledge toward artificial intelligence in medicine: benefits and drawbacks. Heliyon. (2023) 9:e14744. doi: 10.1016/j.heliyon.2023.e14744 37035387 PMC10073828

[30] Lukic A Kudelic N Anticevic V Lazic-Mosler E Gluncic V Hren D. First-year nursing students’ attitudes towards artificial intelligence: Crosssectional multi-center study. NURSE Educ Pract. (2023) 71:103735. doi: 10.1016/j.nepr.2023.103735 37541081

[31] Stewart J Lu J Gahungu N Goudie A Fegan PG Bennamoun M. Western Australian medical students’ attitudes towards artificial intelligence in healthcare. PLoS One. (2023) 18:e0290642. doi: 10.1371/journal.pone.0290642 37651380 PMC10470885

[32] Allam AH Eltewacy NK Alabdallat YJ Owais TA Salman S Ebada MA. Knowledge, attitude, and perception of Arab medical students towards artificial intelligence in medicine and radiology: a multi-national cross-sectional study. Eur Radiol (2023). doi: 10.1007/s00330-023-10509-2 (Epub ahead of print). PMC11213794 38150076

[33] Shinners L Grace S Smith S Stephens A Aggar C. Exploring healthcare professionals’ perceptions of artificial intelligence: piloting the Shinners artificial intelligence perception tool. Digit Health. (2022) 8:205520762210781. doi: 10.1177/20552076221078110 PMC8832586 35154807

[34] Grassini S. Development and validation of the AI attitude scale (AIAS-4): a brief measure of general attitude toward artificial intelligence. Front Psychol. (2023) 14:1191628. doi: 10.3389/fpsyg.2023.1191628 37554139 PMC10406504

[35] Zheng B Wu M Zhu S Zhou H Hao X Fei F. Attitudes of medical workers in China toward artificial intelligence in ophthalmology: a comparative survey. BMC Health Serv Res. (2021) 21:1067. doi: 10.1186/s12913-021-07044-5 34627239 PMC8501607

[36] Hinkin TR. A brief tutorial on the development of measures for use in survey questionnaires. Organ Res Methods. (1998) 1:104–21. doi: 10.1177/109442819800100106

[37] Syed W Basil A Al-Rawi M. Assessment of awareness, perceptions, and opinions towards artificial intelligence among healthcare students in Riyadh, Saudi Arabia. Medicina. (2023) 59:828. doi: 10.3390/medicina59050828 37241062 PMC10221309

[38] Kansal R Bawa A Bansal A Trehan S Goyal K Goyal N. Differences in knowledge and perspectives on the usage of artificial intelligence among doctors and medical students of a developing country: a cross-sectional study. Cureus. (2022) 14:e21434. doi: 10.7759/cureus.21434 35223222 PMC8860704

[39] Truong NM Vo TQ Tran HTB Nguyen HT Pham VNH. Healthcare students’ knowledge, attitudes, and perspectives toward artificial intelligence in the southern Vietnam. Heliyon. (2023) 9:e22653. doi: 10.1016/j.heliyon.2023.e22653 38107295 PMC10724669

[40] Sit C Srinivasan R Amlani A Muthuswamy K Azam A Monzon L. Attitudes and perceptions of UK medical students towards artificial intelligence and radiology: a multicentre survey. Insights Imag. (2020) 11:14. doi: 10.1186/s13244-019-0830-7 PMC7002761 32025951

[41] Hamedani Z Moradi M Kalroozi F Manafi Anari A Jalalifar E Ansari A. Evaluation of acceptance, attitude, and knowledge towards artificial intelligence and its application from the point of view of physicians and nurses: a provincial survey study in Iran: a cross-sectional descriptive-analytical study. Health Sci Rep. (2023) 6:e1543. doi: 10.1002/hsr2.1543 37674620 PMC10477406

[42] Doumat G Daher D Ghanem N-N Khater B. Knowledge and attitudes of medical students in Lebanon toward artificial intelligence: a national survey study. Front Artif Intellig. (2022) 5:1015418. doi: 10.3389/frai.2022.1015418 PMC9668059 36406470

[43] Swed S Alibrahim H Elkalagi NKH Nasif MN Rais MA Nashwan AJ. Knowledge, attitude, and practice of artificial intelligence among doctors and medical students in Syria: a cross-sectional online survey. Front Artif Intellig. (2022) 5:1011524. doi: 10.3389/frai.2022.1011524 PMC9558737 36248622

[44] Castagno S Khalifa M. Perceptions of artificial intelligence among healthcare staff: a qualitative survey study. Front Artif Intellig. (2020) 3:578983. doi: 10.3389/frai.2020.578983 PMC7861214 33733219

[45] Amiri H Peiravi S rezazadeh shojaee S Rouhparvarzamin M Nateghi MN Etemadi MH. Medical, dental, and nursing students’ attitudes and knowledge towards artificial intelligence: a systematic review and meta-analysis. BMC Med Educ. (2024) 24:412. doi: 10.1186/s12909-024-05406-1 38622577 PMC11017500

[46] Sommer D Schmidbauer L Wahl F. Nurses’ perceptions, experience and knowledge regarding artificial intelligence: results from a cross-sectional online survey in Germany. BMC Nurs. (2024) 23:205. doi: 10.1186/s12912-024-01884-2 38539169 PMC10967047

[47] Labrague LJ Aguilar-Rosales R Yboa BC Sabio JB De Los Santos JA. Student nurses’ attitudes, perceived utilization, and intention to adopt artificial intelligence (AI) technology in nursing practice: a cross-sectional study. Nurse Educ Pract. (2023) 73:103815. doi: 10.1016/j.nepr.2023.103815 37922736

[48] Bz W Fg D Mm W. Exploring the opportunities and challenges of implementing artificial intelligence in healthcare: a systematic literature review. Urol Oncol. (2024) 42:48–56. doi: 10.1016/j.urolonc.2023.11.019 38101991

[49] Yu W Zhang Y Xianyu Y Cheng D. Stressors, emotions, and social support systems among respiratory nurses during the omicron outbreak in China: a qualitative study. BMC Nurs. (2024) 23:188. doi: 10.1186/s12912-024-01856-6 38515080 PMC10956170

[50] Murphy K Di Ruggiero E Upshur R Willison DJ Malhotra N Cai JC. Artificial intelligence for good health: a scoping review of the ethics literature. BMC Med Ethics. (2021) 22:14. doi: 10.1186/s12910-021-00577-8 33588803 PMC7885243

[51] Bærøe K Gundersen T Henden E Rommetveit K. Can medical algorithms be fair? Three ethical quandaries and one dilemma. BMJ Health Care Inform. (2022) 29:e100445. doi: 10.1136/bmjhci-2021-100445 PMC8996015 35396245

[52] Hobensack M von Gerich H Vyas P Withall J Peltonen LM Block LJ. A rapid review on current and potential uses of large language models in nursing. Int J Nurs Stud. (2024) 154:104753. doi: 10.1016/j.ijnurstu.2024.104753 38560958

[53] Rony MKK Kayesh I Bala SD Akter F Parvin MR. Artificial intelligence in future nursing care: exploring perspectives of nursing professionals—a descriptive qualitative study. Heliyon. (2024) 10:e25718. doi: 10.1016/j.heliyon.2024.e25718 38370178 PMC10869862

